# Thinking Well: A randomised controlled feasibility study of a new CBT therapy targeting reasoning biases in people with distressing persecutory delusional beliefs

**DOI:** 10.1016/j.jbtep.2015.02.007

**Published:** 2015-09

**Authors:** Helen Waller, Richard Emsley, Daniel Freeman, Paul Bebbington, Graham Dunn, David Fowler, Amy Hardy, Elizabeth Kuipers, Philippa Garety

**Affiliations:** a King's College London, The Institute of Psychiatry, Psychology & Neuroscience, UK; b Centre for Biostatistics, Institute of Population Health, The University of Manchester, Manchester Academic Health Science Centre, UK; c Department of Psychiatry, Oxford University, Oxford, UK; d Mental Health Sciences Unit, Faculty of Brain Sciences, University College London, UK; e Department of Psychology, University of Sussex, Brighton, UK

**Keywords:** Psychosis, Reasoning, CBT, Belief flexibility

## Abstract

**Background and objectives:**

Delusional beliefs with persecutory content are common in psychosis, but difficult to treat. Interventions targeting hypothesised causal and maintaining factors have been proposed as a way of improving therapy. The current study is a feasibility randomised controlled trial of the ‘Thinking Well (TW)’ intervention: This novel approach combines the recently developed Maudsley Review Training Programme (MRTP), with additional, focussed cognitive-behavioural therapy sessions.

**Methods:**

31 participants with distressing persecutory delusions and schizophrenia spectrum disorders were randomised to TW or to treatment as usual in a 2:1 ratio. Participants completed outcome assessments at 0 (baseline), 1 (post-MRTP), 6 (post-TW) and 8 (follow-up) weeks. Key outcomes included belief flexibility, paranoia, and delusional conviction and distress. Participants allocated to TW completed the MRTP package and four CBT sessions with a clinical psychologist.

**Results:**

Recruitment proved feasible. Participants reported the intervention was relevant and had resulted in positive changes in thinking and mood, which they could use in everyday life. Treatment effects were moderate-large for key outcomes including belief flexibility, paranoia conviction and distress. The additional TW sessions appeared to confer benefits over MRTP alone.

**Limitations:**

Assessments were not carried out blind to treatment condition. Recruitment was opportunistic, from an identified pool of research participants. Finally, a few participants had already completed the MRTP as part of a previous study.

**Conclusions:**

The TW intervention appears to be feasible and acceptable to participants, and the effects of treatment are promising. A fully powered randomised controlled trial of the intervention is warranted.

## Highlights

• The Thinking Well intervention was feasible to deliver and feedback was positive.
• Analyses suggest large effects post intervention, with confidence intervals favouring the intervention.
• Effects on state paranoia were not sustained; future work should focus on maintenance.
• A larger study powered to detect changes in key outcomes is warranted.

## 1 Introduction

It was quite simple. I learnt to slow down and think carefully about the situation. In the future I will be very hesitant about coming to a fixed conclusion. ‘Sandra’.

Psychosis is a heterogeneous condition, with a range of symptoms maintained by potentially different causal mechanisms. It has consequently been argued that its treatment will be made more effective by focussing on single symptoms and developing interventions to target the mechanisms maintaining them (Freeman, 2011; Garety & Freeman, 2013).

Persecutory delusions are one of the most common psychotic symptoms in schizophrenia-spectrum disorders, present in 50% of people so diagnosed (and over 70% at first episode; Sartorius, Jablensky, & Korten, 1986; Coid et al., 2013). They are highly distressing, often acted upon, and increase the risk of hospitalisation and suicide (Freeman & Garety, 2006; Freeman & Garety, 2014). Existing treatments, whether pharmacological or psychological, are limited in their effects and thus call for significant improvement (Freeman & Garety, 2014; Leucht et al., 2013; Turner, van der Gaag, Karyotaki, & Cuijpers, 2014).

Reasoning biases are central to persecutory delusions: there is robust evidence that such biases contribute to both development and maintenance of delusional beliefs (see Garety & Freeman, 2013, for review). In particular, it has been proposed that limited belief flexibility and data gathering influence the appraisal of disturbing anomalous experiences and adverse events, encouraging a rapid acceptance of implausible ideas, without generating and considering alternative explanations (Garety et al., 2005). Reasoning is therefore a promising intermediary target in treating persecutory delusions. Accordingly, systematic attempts to modify reasoning biases have begun to appear in the literature. Moritz and colleagues, in particular, have pioneered group-based *metacognitive training* (MCT). This provides education and group exercises for ameliorating a range of cognitive biases associated with psychosis, including reasoning. A number of small trials have shown encouraging results for delusion change (see review, Moritz et al., 2014). However, the two largest randomised controlled trials of MCT did not show changes in reasoning or improvements in moderately severe delusions (Moritz et al., 2013; van Oosterhout et al., 2014). Especially for more severe delusions, individualised approaches may be more effective in helping to engender belief flexibility or ‘sow the seeds of doubt’ (Moritz et al., 2014). Building on the work of Moritz and colleagues, we have developed a new individually-delivered brief intervention that targets intensively the specific reasoning mechanisms hypothesised to maintain persecutory delusions.

Our initial work aimed first to establish ‘proof-of-concept’ experimental evidence that targeting the hypothesised reasoning mediators would induce change in paranoia and delusional conviction. Our first study tested the hypothesis that a brief intervention specifically targeting the JTC reasoning bias would improve delusions held with high conviction (Ross, Freeman, Dunn, & Garety, 2011). In comparison to an attention control condition, participants who completed the reasoning training showed a significant increase in data gathering on a reasoning task, and there were small, albeit non-significant, increases in belief flexibility and delusional conviction. We next embedded this intervention in the Maudsley Review Training Programme (Waller, Freeman, Jolley, Dunn, & Garety, 2011). This brief interactive computerised intervention aims to provide education on reasoning biases (belief inflexibility and jumping to conclusions), and then, in five tasks, train participants to employ a number of strategies aimed at identifying and reducing these biases. Three of the tasks include materials designed to trigger paranoid thinking styles, in order to elicit ‘hot cognitions’ and to teach strategies likely to generalise to participants' own experiences and paranoid beliefs. We tried to encourage participants to become more aware of their reasoning processes, to help them to identify and, where appropriate, inhibit rapid, automatic reasoning (‘type one’ reasoning), and to assist their engagement in more analytical or controlled reasoning (‘type two’) (Evans, 2008; Freeman, Lister, & Evans, 2014). Results from an uncontrolled case series (n = 13) with severe delusions were promising, with significant improvements in both reasoning and delusions (Waller et al., 2011). Finally, we conducted a larger proof-of-concept experiment in a group with high conviction and distressing delusions with paranoid content (N = 101). Comparing the Maudsley Review Training Programme with an attention control condition in a randomised controlled design demonstrated significant improvements in both reasoning and persecutory delusions. It also indicated that the improved outcomes for paranoia were mediated by changes in belief flexibility; in particular the recognition that one's judgements may sometimes be mistaken and that alternative explanations might be available (Garety et al., 2014).

We therefore have proof-of-concept evidence that belief flexibility and data gathering can be improved in people with severe delusions, and that changes in reasoning (in particular, belief flexibility) mediate improvement in delusions. However, a more intensive therapeutic intervention using trained therapists is needed to establish longer-term, more broadly based and clinically important benefits, including larger effects on delusional conviction, preoccupation and distress.

The next step is to move from this experimental work to the therapeutic realm. We have developed a new therapy approach; the ‘Thinking Well’ intervention. This is a brief therapy that builds on the Maudsley Review Training Programme (MRTP) by combining it with four subsequent reasoning-focussed CBT therapy sessions. These are delivered by trained therapists, and are tailored to the person's specific paranoid delusional beliefs, working towards a selected personal goal that is difficult to achieve because of the delusions. As before, it is delivered individually, and suitable for people with moderate to severe delusions.

The current study reports a feasibility randomised controlled trial of the Thinking Well intervention. Its aims were to:

1. Test the feasibility and acceptability of the intervention;
2. Provide initial estimates of the effects of the intervention on belief flexibility and paranoia (distress, conviction and preoccupation);
3. To undertake a preliminary examination of whether the therapy sessions augment the effects of the MRTP alone.

## 2 Methods

### 2.1 Participants

31 participants with persistent, stable persecutory delusions were recruited from adult community mental health teams in three large mental health Trusts in London, between October 2010 and November 2011. The sample size of approximately 30 was chosen *apriori* in order to assess feasibility and conduct preliminary statistical analyses. Power calculations were not conducted at this pilot stage. Inclusion criteria were: a diagnosis of Schizophrenia Spectrum Psychosis (ICD-10, F20-29); a current delusion with persecutory content, assessed using SCAN (Wing et al., 1990), rated as distressing (>0) on a visual analogue scale, and held with at least 50% conviction; aged 18–65 at study entry; and a sufficient grasp of English to complete measures and participate in the intervention. Exclusion criteria were: a primary diagnosis of alcohol or drug dependency; an organic syndrome; a learning disability; a major psychotic relapse or crisis in the last three months. Participants had all previously consented to take part in a previous research study, the ‘Cognitive Mechanisms of Change in Delusions Study’. This comprised two experimental studies: one involved completing the MRTP or an attention-control condition (see Garety et al., 2014); the other involved exposure to an anxiety provoking or control condition with no therapeutic elements (Freeman et al., 2014). Of the 31 participants, six people had previously received the MRTP intervention; these were all required to meet the inclusion criterion for the continued presence of high conviction distressing delusions. Of the six, three were randomly allocated to the intervention group and therefore completed the MRTP for a second time, and three were allocated to the treatment as usual control.

**Fig. 1:**
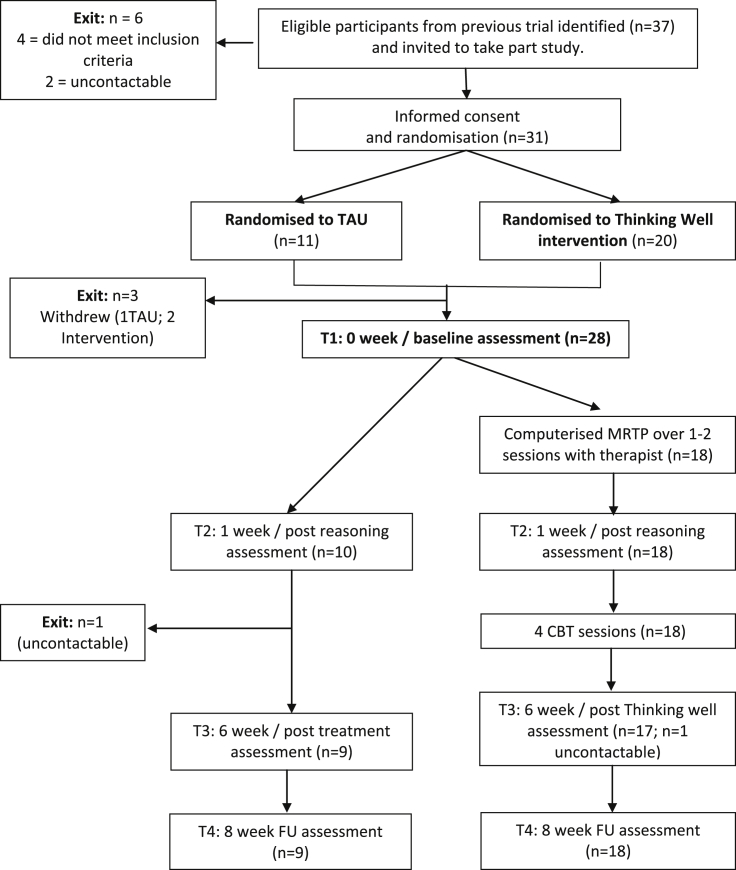
Consort diagram.

A total of 37 potential participants were identified and invited to participate, with the goal of reaching our recruitment target of 30, on the assumption that not all of these would consent to take part. Of these, four did not meet the inclusion criteria above, two were uncontactable and 31 consented. Three further participants withdrew from the study following randomisation, but before completion of baseline clinical assessments and before beginning the intervention (see Fig. 1).

### 2.2 Design

The study was a pilot randomised controlled trial, which was an amendment to a programme of studies (ISRCTN: 59501939; ethics reference: 07/H0803/140; amendment for this study: June, 2010). Participants were randomised by the study coordinator, using an online tool (www.sealedenvelope.com), to either the ‘Thinking Well’ intervention or treatment as usual (TAU). The study coordinator was unblind to treatment allocation and informed participants of the outcome of the randomisation. Randomisation was at the point of consent, using a 2:1 ratio of experimental to control participants.

Study assessments were completed by research workers, who were not blind to treatment group, at four time points: Time 1 (0 weeks; baseline assessment), Time 2 (1 week; post-MRTP), Time 3 (6 weeks, post-combined Thinking Well intervention) and Time 4 (8 weeks; two week follow-up).

### 2.3 Interventions

#### 2.3.1 Treatment as Usual (TAU)

Participants randomised to the TAU group continued to receive their usual care in community mental health services. This included meetings with a care coordinator and less frequent meetings with a psychiatrist, to discuss day to day management of their social, physical and mental health needs. They did not receive any psychological therapy focussed on their delusions for the period of the study.

#### 2.3.2 Thinking Well Intervention (TW)

Participants randomised to the intervention group met with a Clinical Psychologist, expert in CBT with psychosis, to complete the therapy. First, participants completed the Maudsley Review Training Programme (MRTP) over either one or two sessions, depending on participant preference. This was followed by four individualised therapy sessions. The MRTP is described in detail elsewhere (Waller et al., 2011), but, in short, it is a computerised programme aiming to describe and normalise reasoning biases (limited belief flexibility and data gathering) and to teach people how to identify and change these, through the use of five training tasks. The tasks included learning to slow down and look for more information, generating alternative, less upsetting explanations for experiences, and thinking about how mood and past experiences impact on thinking. All tasks were designed to be interactive and engaging, and included simple puzzles, video recordings and short film clips. The focus in both training and homework exercises was on exploring how people come to decisions and make sense of their everyday experiences. Participants complete the computer programme together with the therapist, who discusses key points and reflects on their comments. At the end of the programme, participants are shown how to use tailored thought records with ‘an upsetting thought’, in order to consider whether there could be any chance that they have formed an (over-rapid) view, which might be mistaken. This aims to enhance the participant's belief flexibility and to recruit greater use of type two, reflective reasoning (Evans, 2008). Following completion of the MRTP, participants then met with the therapist to complete 4 h-long, weekly CBT-based therapy sessions. These sessions aimed to help participants apply the learning from the MRTP to their own upsetting beliefs, in the context of working towards a participant-chosen goal. For example, one person wished to be able to get to the local shops more often, which was hindered by a paranoid belief of being targeted by a government agency.

All sessions began with a review of the past week and completion of weekly ratings of reasoning and progress towards their chosen goal. The therapist and participant also discussed any reasoning biases they had noticed over the previous week, including feedback from the therapist, in order to normalise this. In the first session, the therapist worked with the participant to develop a basic formulation of the pattern of interpreting situations and resulting thoughts, feelings and behaviours. They then introduced the idea of stopping, slowing down and thinking through whether there could be another way of looking at the situation, using the learning and training tips from the MRTP. The homework from the previous session was then discussed and any difficulties were problem-solved. If the participant had not been able to complete the homework, they were encouraged to complete a thought record during the session, thinking through an ‘upsetting situation’ from the previous week. Participants were gently encouraged to think through all of the learning and strategies (tips) from the MRTP in relation to the upsetting thought described. In the case where they did not feel that the tips applied (where conviction remained high and flexibility low) there was an opportunity to discuss ways of feeling better e.g. pleasurable activities, talking to a friend, distraction, relaxation. Personalised coping cards were made for the participant to take away and use between sessions. In the final session, any changes in reactions, feelings and behaviours were reviewed in relation to the participant's chosen goal and main upsetting belief and the therapist talked through relapse prevention strategies. They were given a copy of all handouts to take away.

### 2.4 Measures

All measures were assessed at each of the four time points, with the exceptions of the Scale for the Assessment of Positive Symptoms (SAPS), used only at baseline, and participant feedback, which was elicited from those in the intervention group at post-intervention. The primary outcomes, where relevant, were all in relation to participants' main, most strongly held, delusional belief.

#### 2.4.1 Positive symptoms

The SAPS (Andreasen, 1984) is a semi-structured interview for assessing positive symptoms in four areas: hallucinations, delusions, bizarre behaviour and positive formal thought disorder. The presence of symptoms in each area is rated from 0 (absent) to 5 (severe). Positive symptoms were assessed at baseline only to provide a summary of clinical characteristics of the sample. The scale has good psychometric properties (Andreasen, 1984).

#### 2.4.2 Belief flexibility

Belief flexibility was assessed using two items previously employed extensively in large scale studies (e.g. Garety et al., 2014; So et al., 2012). The Explanations of Experiences Assessment (Freeman et al., 2004) was used to assess the number of alternative explanations for a person's experiences at each time point. Participants were asked whether there was anything else that could explain the evidence given for their delusional belief (‘*Are there any other reasons—other than [state main belief] that could possibly account for these experiences even if you think they are very unlikely?*’). The number of distinct explanations given is recorded. One item from the Maudsley Assessment of Delusions Schedule (MADS, Wessely et al., 1993) was used to assess the participants' acceptance of the possibility of being mistaken about their persecutory belief by asking, after a full description of the belief and the grounds for it had been elicited, ‘*When you think about it now*, *is it at all possible that you are mistaken about this?*’ Responses to this question were rated as a percentage (0–100% possibility of being mistaken).

#### 2.4.3 State paranoia

A series of five visual analogue scales (VAS) items were used to assess state paranoia taken from Green et al.'s (2008) Paranoid Thought Scales (‘I am deliberately being harmed or upset’, ‘I am being followed’, ‘there is a conspiracy against me’, ‘I am being persecuted’, ‘I am being laughed at behind my back’). These items were selected as having both high loadings on the persecution or reference subscales, and also as representative of a range of key paranoid concerns; they have good internal reliability (Cronbach's alpha = 0.86 (Freeman, Dunn, Fowler, et al., 2013; Freeman, Dunn, Garety, et al., 2013). In response to each item, participants were asked to rate, ‘how you have been feeling *over the last 15 min*’ in response to each item, ranging from 0 (not at all) to 100 (totally). The mean of the five items is calculated at each time point to provide a total paranoia score (Freeman, Dunn, Fowler, et al., 2013; Freeman, Dunn, Garety, et al., 2013).

#### 2.4.4 Delusional conviction

Conviction was rated using a visual analogue scale asking participants to state how much they believe the main belief is true, ‘right now’, ranging from 0 (believe not at all) to 100% conviction (believe absolutely).

#### 2.4.5 Delusional distress and preoccupation

Participants were ask to rate how much, ‘right now’, their main belief occupies their mind and upsets them, both on a 100 point VAS scale ranging from 0 (not at all) to 100 (totally).

#### 2.4.6 Participant feedback

At post-intervention participants in the intervention group were asked to provide feedback on their experiences of the sessions, using a semi-structured interview. Feedback was elicited on participants' experiences of the MRTP training package and therapy sessions, working with the therapist, how helpful and relevant the sessions were for their problems and whether they learnt any new skills to use in future. Ideas for improvement and on any unhelpful aspects of the sessions were also sought.

### 2.5 Analysis

The acceptability and feasibility of the Thinking Well Intervention was assessed through recruitment, drop-outs and feedback interviews with participants in the intervention group. Feedback interviews are summarised descriptively.

To estimate intention-to-treat effects (i.e. differences between the two groups), a series of analyses were conducted in STATA (v13.1, StataCorp, 2013). The analysis used separate linear regression models for outcome variables at times 2, 3 and 4, with a main effect of treatment and adjusted for baseline values of the outcome measure. For the binary outcomes, we used exact logistic regression, which is more appropriate for small samples and gives an exact p-value. We report Cohen's D standardised effect sizes and their corresponding bootstrapped 95% confidence intervals (CIs), based on unadjusted mean differences and the pooled standard deviation at each time point. As recommended in guidelines for good practice for the analysis of pilot studies (Lancaster, Dodd, & Williamson, 2004), the focus of the results is on the estimates of the treatment effects and the corresponding 95% CIs for the mean difference. Since the study was not designed or powered for testing differences between groups, the p values are reported for completeness but note that the CIs of the treatment effect estimates are of greater relevance.

## 3 Results

A summary of clinical and demographic information for participants in both groups is displayed in Table 1.

Recruitment to the intervention was successful, and there were few drop outs (3 out of 31). The two people who withdrew from the TW group did so after randomisation, but prior to starting the intervention. The remaining 18 participants allocated to the intervention group completed the entire intervention. The majority of participants completed assessments at all time points, with the exception of one person from the TAU group, who was uncontactable at times three and four, and another participants from the TW group who was uncontactable at time three only. No adverse events were recorded for any participants during the study period.

### 3.1 Statistical analysis

Summary statistics for all outcomes across time points and conditions are presented in Table 2. The main ITT analyses, comprising separate linear regression models for outcome variables at times 2, 3 and 4, are presented in Table 3 . Inspection of the effect sizes and confidence intervals suggests that there were improvements in key outcome measures for those in the Thinking Well group, relative to the TAU group. The regression coefficients (i.e. the treatment effects) and the standardised effect sizes (Cohen's D) are generally numerically considerably higher in the immediate post-Thinking Well assessments (time 3), than at the post-MRTP (time 2) and follow-up assessment points (time 4) (Table 3).

### 3.2 Participant feedback

Overall feedback was positive. Of the 17 respondents, approximately two thirds reported a positive experience with the TW intervention, whilst one third reported a more neutral experience. Specific examples and illustrative quotations of the feedback are summarised in Table 4.

## 4 Discussion

The results of the study are promising, both in terms of the feasibility and acceptability of the Thinking Well intervention, and of the estimated treatment effects. Recruitment to the study ran smoothly, and although two participants withdrew prior to starting therapy, there were no drop outs following commencement of therapy. Feedback from participants was typically positive, suggesting that they had enjoyed the sessions, understood the content, become more reflective and actively learnt new skills, which they had then been able to apply outside the sessions and which had improved their mood and wellbeing.

The *a priori* focus of these results is on the estimates of the treatment effects and the corresponding 95% CIs for the mean difference (Lancaster et al., 2004). The treatment effects immediately after the Thinking Well intervention favoured the intervention condition, and were large. This suggests that at post-intervention the addition of individual Thinking Well therapy sessions may confer additional benefits over MRTP alone. The size of the MRTP effects are consistent with and comparable to those found in our previous, larger (n = 101) proof-of concept study (e.g. standardised effect size (ES) for paranoia: Cohen's D = 0.38 cf 0.36 in Garety et al., 2014); after the combined TW intervention in this pilot study this rises to an effect size of 1.07. Additionally, clinically beneficial effects were seen not only for belief flexibility and state paranoia, but also on distress (after TW ES 1.3), and conviction (ES 0.6). From the perspective of the patient, these are important targets of the therapy, and carry the prospect of underpinning sustained change; our previous briefer interventions did not achieve a comparable change, especially in delusional conviction.

However, the results suggest that the effects post-TW are not fully sustained at follow up: there were lower estimated effects at follow-up in comparison to post-treatment, on all key outcomes including belief flexibility (post TW ES = −1.0; follow-up = −0.4), state paranoia (post TW = 1.1; follow-up = 0.6) and delusional conviction (post TW = 0.6; follow-up = 0.4). There were some suggestions from the participant feedback data on ideas for improvement (n = 4): that the sessions could be more personalised and build in greater support to generalise the skills for use outside the therapy sessions. We are currently extending the intervention from 4 to 8 sessions, and integrating the MRTP and additional Thinking Well sessions into a more intensive and sustained approach to generalisation and homework, with enhanced digital tools for self-management strategies.

Additionally, a small proportion of clients reported that they did not find the intervention (or indeed any type of therapy for some) relevant to their problems. From this small pilot study we cannot identify which patients will benefit. Our previous proof-of-concept experiment suggested that working memory and negative symptoms moderated effects of the intervention on reasoning (Garety et al., 2014). We also know that beliefs about the causes and treatability of one's problems and the relevance of psychological treatment may affect engagement, and consequently outcome (Freeman, Dunn, Garety, et al., 2013; Marcus et al., 2014). An important goal of future research is to clarify the reasons for treatment engagement and treatment effect heterogeneity. Building on our earlier research, we plan in future to examine whether characteristics of the participants (including working memory and negative symptoms) moderate the effects of the intervention on reasoning and also whether beliefs about their problems affects receipt of an adequate dose of treatment-as-intended.

The current pilot research design had limitations, including the lack of blinding of research workers to treatment condition and the opportunistic recruitment procedure, identifying eligible participants from those who had already taken part in a previous study. Six participants had already received the MRTP package, three of whom were allocated to the intervention group and therefore completed the package on two occasions, while three were allocated to the control group. It is unclear what impact this would have had on the results, although going through the package twice may have acted as a booster and might have led to greater improvements. However, all participants were screened before entry and continued meet inclusion criteria, so that all of the current sample had persistent, treatment-resistant delusional beliefs at baseline. We note that there were chance baseline differences in delusions variables between the two groups, and that if these variables are prognostic of outcome, future larger studies should stratify randomisation by these variables.

Overall, we conclude that following modifications aimed at improving the generalizability, maintenance of effects and real-life application of the therapy, the current findings hold promise and justify proceeding to a fully powered RCT.

## Funding

Wellcome Trust project grant (085396).

## Figures and Tables

**Table 1:** Clinical and demographic information: means (SD) and numbers of participants.

	Total randomised sample (n = 31)	Thinking Well group (n = 20)	TAU group (n = 11)	
Age (years)	41.11 (10.56)	39.05 (10.54)	43.00 (10.69)	
Sex:				
Male	22 (71%)	15 (75%)		7 (64%)
Female	9 (29%)	5 (25%)		4 (36%)
Ethnicity				
White British/Irish	13 (41.9%)	7 (35%)		6 (54.5%)
Black Caribbean	8 (25.8%)	6 (30%)		2 (18.2)
Mixed Race	3 (9.7%)	2 (10%)		1 (9.1%)
Black African	3 (9.7%)	2 (10%)		1 (9.1%)
Black other	2 (6.5%)	2 (10%)		
Asian	1 (3.2%)	0 (0%)		1 (9.1%)
Other ethnic group	1 (3.2%)	1 (5%)		
Diagnosis				
Schizophrenia	27 (87.1%)	16 (80%)		11 (100%)
Delusional Disorder	2 (6.5%)	2 (10%)		0 (0%)
Schizoaffective Disorder	2 (4.5%)	2 (10%)		0 (0%)
Length of illness (years)	12.66 (8.62)	10.49 (7.87)		14.91 (8.67)
Medication				
Yes	28 (90.3%)	18 (90%)		10 (90.9%)
No	3 (9.7%)	2 (10%)		1 (9.1%)
Years of education	13.48 (2.71)	14.21 (2.82) (n = 19)		13.50 (3.06) (n = 10)
Marital Status				
Single	25 (80.6%)	17 (85%)		8 (72.7%)
Previously married or cohabiting	5 (16.1%)	2 (10%)		3 (27.3%)
Married	1 (3.2%)	1 (5%)		0 (0%)
Employment Status				
Unemployed	28 (90.3%)	18 (90%)		11 (100%)
Employed	1 (3.2%)	2 (10%)		0 (0%)
Positive Symptoms: SAPS ratings	(n = 28)	(n = 18)		(n = 10)
Hallucinations	2.89 (1.66), range = 0-5	2.44 (1.58)		3.70 (1.57)
Delusions	4.07 (0.60), range = 3-5	4.17 (0.62)		3.90 (0.57)
Bizarre behaviour	0.57 (0.92), range = 0-3	0.56 (0.86)		0.60 (1.08)
Positive formal thought disorder	0.86 (1.11), range = 0-4	1.06 (1.21)		0.50 (0.85)

_Key: TAU = Treatment as usual; SAPS = Scale for the Assessment of Positive Symptoms (_Andreasen, 1984_)._

**Table 2:** Summary statistics at each time point for all outcome measures for each randomised group separately.

Measure	Time	‘Thinking Well’ group				‘Treatment as usual’ group			
		Mean %	SD	Range	N	Mean	SD	Range	N
Belief Flexibility: % participants with alternative explanations	T1	22.2%	–	–	18	0.0%	–	–	10
	T2	33.3%	–	–	18	20.0%	–	–	10
	T3	47.1%	–	–	17	0.0%	–	–	9
	T4	55.6%	–	–	18	11.1%	–	–	9
Belief flexibility: Possibility of being mistaken %	T1	22.61	27.57	0–75	18	22.22	23.20	0–50	9
	T2	27.22	28.40	0–80	18	6.67	10.90	0–25	9
	T3	27.19	31.14	0–100	16	1.25	3.54	0–10	8
	T4	30.29	32.67	0–100	17	16.67	33.07	0–100	9
State paranoia	T1	37.83	20.95	0–79	18	51.00	36.91	0–95	10
	T2	44.17	24.25	0–100	18	52.60	17.38	27–79	10
	T3	29.53	21.45	0–76	17	57.00	32.57	10–100	9
	T4	38.39	28.60	0–98	18	52.89	19.21	25–80	9
Delusional conviction	T1	78.61	23.31	25–100	18	84.44	26.63	25–100	9
	T2	74.17	28.04	25–100	18	88.33	24.24	25–100	9
	T3	55.00	36.36	0–100	17	76.11	33.15	0–100	9
	T4	66.39	32.58	0–100	18	78.89	32.57	20–100	9
Delusional distress	T1	63.89	25.98	20–100	18	81.11	24.21	25–100	9
	T2	69.17	26.30	25–100	18	80.00	21.07	50–100	9
	T3	44.82	32.06	0–100	17	83.57	23.22	40–100	7
	T4	58.33	35.52	0–100	18	75.00	29.47	10–100	9
Delusional preoccupation	T1	56.39	28.53	20–100	18	71.11	28.48	25–100	9
	T2	58.33	26.35	0–100	18	73.33	19.84	50–100	9
	T3	45.71	32.42	0–100	17	70.71	18.13	40–100	7
	T4	51.78	33.03	0–100	18	68.89	32.19	15–100	9

_Key: SD = standard deviation_.

**Table 3:** Effect of experimental group compared to control group on outcome measures at Times 2, 3 and 4.

Measure	Time 2 (post MRTP)			Time 3 (6 weeks/post thinking Well)			Time 4 (8 week follow-up)		
	Effect (SE); 95% CI	p-value	Cohen's D; 95% CI	Effect (SE); 95% CI	p-value	Cohen's D; 95% CI	Effect (SE); 95% CI	p-value	Cohen's D; 95% CI
Belief Flexibility: Alternative explanations	OR = 1.47 (n/a), 1.00; CI = 0.09, 24.36	–	–	OR = 0.17 (n/a), 0.119; CI = 0.00, 1.50	–	–	OR = 0.18 (n/a), 0.249	–	–
Belief Flexibility: Probability mistaken	20.35 (8.23); 3.35, 37.34	0.021	−0.85; −1.47, −0.23	26.05 (10.61); 3.98, 48.12	0.023	−1.01; −1.59, −0.42	12.23 (9.82); −8.09, 32.54	0.226	−0.42; −1.34, 0.51
State Paranoia	−2.03 (7.28); −17.02, 12.96	0.782	0.38; −0.39, 1.16	−13.96 (6.98); −28.93, 0.48	0.057	1.07; −0.07, 2.20	−5.54 (9.91); −26.00, 14.92	0.581	0.56; −0.34, 1.45
Delusional Conviction	−9.90 (8.43); −27.31, 7.50	0.252	0.53; −0.41, 1.47	−17.93 (13.57); −46.00, 10.14	0.199	0.60; −0.31, 1.51	−8.98 (12.19); −34.13, 16.17	0.468	0.38; −0.55, 1.32
Delusional Distress	−2.54 (9.44); −22.03, 16.95,	0.790	0.44; −0.31, 1.18	−23.69 (12.89); −50.50, 3.12,	0.080	1.30; 0.33, 2.26	−7.94 (13.67); −36.16, 20.28	0.567	0.49; −0.31, 1.30
Delusional Preoccupation	−9.82 (9.58); −29.59, 9.96	0.316	0.61; −0.11, 1.34	−20.64 (13.47); −48.65, 7.38,	0.140	0.86; 0.07, 1.64	−17.47 (13.54); −40.42, 15.48	0.366	0.52; −0.39, 1.44

_Key: SE = standard error;__OR = odds ratio; CI = confidence interval_.

**Table 4:** Participant feedback: descriptions and illustrative quotations.

**Positive views (n = 11)**	
General:	‘*Lovely*, *really enjoyed it*’ ‘*Excellent*, *very worth doing*’ ‘*Enlightening*’
Learning new skills:	‘*I learnt my mood can affect my thinking*’ ‘*[I learnt] how to weigh up conclusions*’ ‘*I learnt a different way of thinking*: *don*'*t JTC*; *feel strong and confident*’; ‘*I learnt connections to behaviours and beliefs and mental health - touched me deeply*’
Application of new skills:	‘*Now I look at the evidence – a lot of it was paranoia*; *the way I process my thoughts*’ ‘*I think about the reasons why the police may not be following me – I look at things objectively* ‘*I try not to JTC*: *I assess my mood*’ ‘*It gave me another perspective*; *not look at things as they appear*, *but look from a different angle*.’
Relationship with the therapist:	‘*They showed concern toward me*’ ‘*The way the therapist explained things – it was a good chat*’
Impact on mood and confidence:	‘*I feel a lot stronger – I was feeling dreadful before*’ ‘*Thinking differently makes me feel better*’ ‘*Made me more motivated –it*'*s going to be okay*’ ‘*More relaxed and not so prone to getting tense when I am outdoors*’
**Neutral or negative views (n = 6)**	
General:	‘*It was okay*, *but a bit boring*’ ‘*Same as before with [previous psychologist] but more paperwork*’
Not relevant to personal problems:	‘*No*, *it*'*s not what the doctor prescribes*' ‘*Not really [relevant]*; *some of the questions did not really apply to me*’ ‘*The whole JTC thing is not relevant to me*. *I don*'*t think I JTC*’ ‘*It is not giving any remedy*’
Belief that therapy cannot help:	‘*[I don*'*t think] anything can help*’ ‘*Wasn*'*t really that helpful*; *can*'*t feel good without alcohol*’
**Ideas for improvements (n = 4)**	
Personalising Sessions:	‘*The videos are a bit too vague*; *not very concise and relevant* *Couldn*'*t apply everything to my situation – ok here but if I was at home would be different*’ ‘*Make more personalised and focus on aspects of distress*’ ‘*Tailor more issues*’
Length of Sessions:	‘*[Sessions could be] shorter – 45 min*’ ‘*Longer sessions or more of them*’
